# Effects of Dietary Yucca Schidigera Extract and Oral *Candida utilis* on Growth Performance and Intestinal Health of Weaned Piglets

**DOI:** 10.3389/fnut.2021.685540

**Published:** 2021-05-26

**Authors:** Zhenguo Yang, Yao Wang, Tianle He, Gifty Ziema Bumbie, Liuting Wu, Zhihong Sun, Weizhong Sun, Zhiru Tang

**Affiliations:** Laboratory for Bio-feed and Molecular Nutrition, College of Animal Science and Technology, Southwest University, Chongqing, China

**Keywords:** yucca schidigera extract, *Candida utilis*, occludin, β-definsin-2, microflora

## Abstract

Weaning piglets experienced the transformation from breast milk to solid feed and present the proliferation of pathogens, the presence of diarrhea, poor growth performance and even death. Plant extracts and probiotics have certain potential in improving animal growth performance, antioxidant capacity and immune function. The purpose of this study was to explore the effects of dietary yucca schidigera extract (YSE) and oral *Candida utilis* (CU) on growth performance and intestinal health weaned piglets. According to a 2 × 2 factorial design with the main factors being CU (orally administered 1 mL of 0.85% saline with or without CU; fed basal diet with or without 120 mg/kg YSE), forty 28 d healthy weaned piglets were randomly allocated into four groups of 10 barrows each: (1) piglets fed basal diet and orally administered 1 mL of 0.85% saline (CON); (2) piglets fed basal diet and orally administered 1 mL 1 × 10^9^ cfu/mL *C. utilis* in 0.85% saline (CU); (3) piglets fed the basal diet containing YSE (120 mg/kg) and orally administered 1 mL of 0.85% saline (YSE); (4) Piglets fed the basal diet containing 120 mg/kg YSE and 1 mL 1 × 10^9^ cfu/mL *C. utilis* in 0.85% saline (YSE+CU). This study lasted 28 days and evaluated the effects of dietary YSE and oral CU on growth performance, immunity, antioxidant function, ileal morphology, and intestinal microflora in weaned piglets. Dietary YSE increased ADG, the spleen and lymph node indexes, serum GLU, BUN, T-SOD, T-AOC, CAT concentrations, ileal villus height and villus height/crypt depth, jejunal occludin, and β-definsin-2 concentrations and ileal occludin concentration in weaned piglets (*P* < 0.05); decreased the diarrhea rate and mortality, rectal pH and urine pH, the BUN and MDA concentrations, crypt depth (*P* < 0.05); improved the diversity of cecal microflora. Orally CU increased ADG, and ADFI, the T-SOD, T-AOC, and CAT activity, ileal villus height, villus height/crypt depth, jejunum occludin, and β-definsin-2 concentrations (*P* < 0.05); reduced the diarrhea rate and mortality, urine pH, the BUN and MDA concentrations, crypt depth (*P* < 0.05); improved the diversity of cecal microflora. Dietary YSE and orally CU increased the T-SOD, T-AOC, and CAT activity, villus height/crypt depth, jejunal occludin concentration; reduced the diarrhea rate of weaned piglets by 28%, gastric pH, ileal pH, cecal pH and urine pH, MDA, crypt depth; improved the diversity of cecal microflora. YSE and CU could improve the growth performance, reduce the diarrhea rate, improve intestinal health, and increase the diversity and abundance of cecal microflora in weaned piglets and expected to be used as antibiotics alternative feed additives in the production of weaned piglets.

## Introduction

The transformation of weaned piglets from breast milk to solid feed will cause intestinal flora disorder, and then lead to diarrhea, growth performance decline, and even death of piglets, and finally bring huge economic losses to animal husbandry. Therefore, the use of active ingredients in weaned piglets can protect the stability of gastrointestinal microorganisms, prevent or slow down diarrhea, improve growth performance and promote the development of antibiotics alternative feed additives. Many studies have pointed out that plants and their extracts play an important role in promoting animal growth, enhancing immunity and maintaining animal health (1–4). YSE is a natural plant extract, which is generally recognized as safe (GRAS), so it can be used in food, cosmetics, pharmaceutical and feed industry. Studies have confirmed that YSE can improve animal feed intake, feed conversion efficiency, growth rate and maintain animal health (5, 6), mainly because YSE has a significant effect on anti-inflammation, antibacterial and enhancing immunity in livestock and poultry (7, 8). Probiotics have attracted wide attention because of their enhancement of animal immunity and effective defense against the invasion of pathogenic bacteria. CU is a kind of forage yeast, which is proved to be a kind of microorganism rich in cell protein, which can improve the balance of intestinal microecology and is beneficial to the growth of the host (9). However, the research on YSE and CU in weaned piglets is insufficient, which limits their application in production. This paper explores the effects of YSE and CU on growth performance and intestinal health and their probiotic mechanism and provided an experimental basis for the application of YSE and CU.

## Methods and Materials

### Yeast, Materials, and Reagents

YSE with an active ingredient content of 60% was purchased from Xi'an Lutian Biotechnology Co., Ltd. (Xi'an, China). CU was purchased from the Deutsche Sammlung von Mikroorganismen und Zellkulturen (No. DSM 2361).

YPD culture medium comprised 2% (w/v) glucose, 1% (w/v) yeast powder, 2% (w/v) peptone, 2% (w/v) agar, and was adjusted to pH 6.0 and sterilized for 15 min at 115°C.

The activities or contents of glucose (GLU), total cholesterol (T-CHO), Aspartate aminotransferase (AST), Alanine transaminase (ALT), blood urea nitrogen (BUN), total superoxide dismutase (T-SOD), total antioxidant capacity (T-AOC), malondialdehyde (MDA), catalase (CAT), acetylcholinesterase (A-CHE) were purchased from Nanjing Jiancheng Bioengineering Institute (Nanjing, China). Power Fecal DNA Isolation Kit was purchased from Germany Qiagen reagent company (Dusseldorf, Germany).

The rabbit-anti rat β-definsin-2 (ab178728, 1:1000) and the rabbit-anti rat occludin (ab31721; 1:1000) were purchased from Abcam (Cambridge, MA, USA). The rabbit-anti rat β-actin (5125S, 1:1000) was purchased from CST (Danvers, USA). The F(ab)_2_ of goat-anti rabbit Ig was purchased from Fantibody (FAB127288, 1: 2500).

### Experimental Design, Animals, and Diets

The experiment was established as a 2 × 2 factorial design with the main factors being *C. utilis* (orally administered 1 mL of 0.85% saline with or without *C. utilis;* fed basal diet with or without 120 mg/kg YSE). Forty 28-day-old weaned piglets (Rongchang × Landrace × Large white) with similar parity and body weight (7.51 ± 0.54 kg) were randomly divided into 4 treatments of 10 barrows each: (1) piglets fed basal diet and orally administered 1 mL of 0.85% saline (CON); (2) piglets fed basal diet and orally administered 1 mL 1 × 10^9^ cfu/mL *C. utilis* in 0.85% saline (CU); (3) piglets fed the basal diet containing YSE (120 mg/kg) and orally administered 1 mL of 0.85% saline (YSE); (4) Piglets fed the basal diet containing 120 mg/kg YSE and 1 mL 1 × 10^9^ cfu/mL *C. utilis* in 0.85% saline (YSE+CU).

The ingredients and the compositions of the basal diet are formulated according to the NRC (2012) recommendations (Table 1). Piglets were fed at 08:00, 12:00, and 18:00, and kept in 4 mechanically ventilated and temperature-controlled (30 ± 1.2°C) 30 m^2^ room. Each piglet was kept in individual pen (1.5 m length × 0.5 m width × 0.8 m height). Food and water were provided *ad libitum*. All experimental procedures were approved by the License of Experimental Animals (SYXK 2014-0002) of the Animal Experimentation Ethics Committee of Southwest University, Chongqing, China.

**Table 1:** The ingredients and nutritional levels of diets (DM basis, %).

**Ingredients**	**Content**	**Nutrition level**	**Content**
Corn	62.90	DE (MJ/kg)	13.80
Soybean meal	19.97	CP (%)	16.42
Fish meal	2.40	Ca (%)	0.72
Whey powder	5.69	CF (%)	2.65
Wheat Bran	5.07	AP (%)	0.37
Soybean oil	0.83	Lys (%)	1.27
Limestone	0.77	Met (%)	0.37
Salt	0.30	Thr (%)	0.75
CaHPO_4_	0.70	Trp (%)	0.22
Sweetener	0.06		
Antioxidant	0.02		
Choline chloride	0.08		
Vitamin premix^a^	0.08		
Trace mineral premix^b^	0.30		
Threonine (Thr)	0.09		
Lysine Hydrochloride (Lys)	0.31		
Methionine (Met)	0.09		
Tryptophan (Trp)	0.34		
Total	100.00		

*AP, available phosphorus;*

a *Vitamin premix provided the following per kg of the diet: vitamin A 2 017 IU, vitamin D 208 IU, vitamin E 14 IU, vitamin K 0.49 mg, pantothenic acid 10.1 mg, riboflavin 3.4 mg, folic acid 0.29 mg, nicotinic acid 29.1 mg, thiamine 1.1 mg, vitamin B_6_ 5.7 mg, biotin 0.06 mg, vitamin B_12_ 0.017 mg.*

b *Trace mineral premix provided the following per kg of the diet: ZnSO_4_·7H_2_O 268 mg, FeSO_4_·7H_2_O 323.33 mg, MnSO_4_·H_2_O 11.54 mg, CuSO_4_·5H_2_O 22.86 mg, KI 14.21 mg, Na_2_SeO_3_ 28.37 mg. ^3^DE was a calculated value, and the others were measured*.

The experimental period lasted for 28 days. Feed intake and incidence of diarrhea in pigs were recorded daily during the entire experimental period. The pigs were weighed on day 0 and day 28 prior to the morning feed.

### Sample Collection

Prior to the morning feed on day 29, five piglets were selected from each group and a 10 mL blood sample was collected. The blood sample was undisturbed for 60 min and centrifuged at 3,500 *g* for 10 min at 4°C to harvest the serum. Serum was stored at −20°C for biochemical analysis and enzyme linked immunosorbent assay (ELISA). After blood sampling, the five pigs with a similar average weight selected from each group were anesthetized with an intravenous injection of sodium pentobarbital (50 mg/kg Basal body weight) and bled by exsanguination. Cardiac, liver, thymus, kidney, spleen, pancreas, mesenteric lymph nodes were sampled and weighed. Then a 2–3 cm of jejunal and ileal tissue was excised from the midpoint of the jejunum, gently rinsed using cold saline, and placed into 10% formalin solution before hematoxylin and eosin (H&E) staining. The jejunal and ileal mucosa were rinsed by cold saline, and the mucosa was scraped gently by a scalpel blade and immediately frozen in liquid N_2_ and stored at −80°C for western blot analysis. The digesta contents of stomach, jejunum, ileum, colon, cecum, rectum and urine were collected for pH measure and he digesta contents of colon were collected for 16S rDNA sequencing.

### Gastrointestinal Tract pH Measure

The digesta contents of stomach, jejunum, ileum, colon, cecum, rectum and urine were measured by pH meter (METTLER TOLEDO, S220, Switzerland).

### Serum Biochemical Index Analysis

The presence of GLU, T-CHO, AST, ALT, BUN, T-AOC, T-SOD, CAT, MDA, and A-CHE in serum were determined using colorimetric methods with a reagent kit according to the manufacturer's instructions (Nanjin Jianchen Institute of Bioengineering, Nanjing, Jiangshu, China).

### H&E Staining

The morphology of the ileum was analyzed by H&E staining as reported by Wang et al. (10) Sliced samples were viewed under an optical microscope (Carl Zeiss Inc., Oberkochen, Bayern, Germany). Each sample photographed Five pictures and five fields in each picture were used to analyze villus height and crypt depth using image analysis software (Intronic GmbH & Co., Rothenstein, Berlin, Germany).

### Western Blot Analysis

About 100 mg jejunal and ileal mucosa was homogenized in 1 mL RIPA buffer [50 mM Tris-base, 1.0 mM ethylene diamine tetraacetic acid (EDTA), 150 mM NaCl, 0.1% sodium dodecyl sulfate (SDS), 1% Tritox-100, 1% sodium deoxycholate, and 1 mM phenylmethylsulfonyl fluoride (PMSF)] and separated by SDS-polyacrylamide gel electrophoresis (SDS-PAGE). The proteins were transferred to a polyvinylidene fluoride (PVDF) membrane by the semi-dry transfer method. The PVDF membranes were blocked in a blocking buffer overnight at 4°C, then incubated in blocking buffer with rabbit-anti rat β-definsin-2, β-actin, occludin and incubated in blocking buffer with F(ab)_2_ of goat-anti rabbit Ig labeled with horseradish peroxidase and with diluted in phosphate buffered saline solution. The PVDF membrane was soaked in a chemiluminescent liquid (Millipore, Massachusetts, USA). Pictures were photographed using a Chemiluminescence Imaging System (Bio-Rad).

### The 16S rDNA Sequencing and Analysis of Cecum Microflora

The total DNA of cecum contents was extracted with the MOBIO Power Fecal DNA Isolation Kit and sent to Chengdu Luoning Biological Technology Co for sequencing and analysis of microbial 16S rDNA fragments. The specific primers with Barcode were synthesized to amplify the 16S rDNA V4 region of the sample using the primers 515F (5′-GTGCC AGCMG CCGCG GTAA-3′) and 806R (5′-GGACT ACHV GGGTW TCTAAT-3′). Three replicates were performed for each sample, and the recovered products of PCR were detected and quantified with Qubit 2.0. The corresponding proportion was mixed according to the sequencing quantity requirements of each sample. the library was constructed by Illumina's TruSeq DNA PCR-Free Sample Prep Kit. Illumina's MiSeq Reagent Kit v2 was used for MiSeq sequencing. PE reads obtained from Miseq sequencing were spliced with FLASH (https://ccb.jhu.edu/software/FLASH/). Data filtering was completed after the removal of low-quality bases and contaminated sequences of joints. The samples were then analyzed with UPARSE (11).

### Data Calculation and Statistical Analysis

The average daily feed intake, average daily weight gain and feed conversion rate (FCR), diarrhea incidence and organ index were calculated according to the following formula: Average daily gain (ADG) (g/d) = (final weight-initial weight) (g)/days (d) (Equation 1); Average daily feed intake (ADFI) (g/d) = total feed intake (g)/days (d) (Equation 2); Feed/Gain = average daily weight gain (g)/average daily food intake (g) (Equation 3); Diarrhea incidence (%) = the number of pigs with diarrhea/(the number of pigs × test days) × 100% (Equation 4); The incidence of diarrhea (%) = the number of diarrhea × days/(number of piglets × days of trial) × 100% (Equation 5); Organ index (g/kg) = organ wet weight (g)/pig live weight (kg) (Equation 6).

Data were analyzed by two-way with CU (2 levels) and YSE (2 levels) analysis of variance using the GLM procedure (SAS Institute 222 Inc.; Cary, NC, USA). The values presented in the tables represent means and pooled SEMs. The Student-Neuman-Keuls test was performed to identify differences among groups. Significance was set at *P* < 0.05.

## Results

### Effects of Dietary YSE and Oral CU on Growth Performance of Weaned Piglets

The effects of oral YSE and CU on the growth performance of weaned piglets were shown in Table 2. Piglets dietary administered YSE increased the final weight and ADG (*P* < 0.05), and tended to reduce the Feed/Gain (*P* = 0.087), and had no significant effect on ADFI (*P*> 0.05). Piglets orally administered CU increased final weight, ADG, and ADFI (*P* < 0.05), didn't affected Feed/Gain (*P* > 0.05). Piglets orally administered CU or dietary administered YES decreased the diarrhea rate and mortality. Piglets orally administered CU and dietary administered YSE reduced the diarrhea rate as much as 28%. There was no the interaction effect on final weight, ADG, and ADFI between YSE and CU (*P* > 0.05), and there was a significant trend in the interaction effect on the Feed/Gain (*P* = *0.057*).

**Table 2:** Effects of dietary YSE and oral CU on growth performance in weaned piglets.

**Items**	**Treatments**				**SEM**	***P*****-value**		
	**CON**	**CU**	**YSE**	**CU+YSE**		**YSE**	**CU**	**CU × YSE**
Initial weight (kg)	7.69	7.55	7.62	7.76	0.12	0.563	0.991	0.253
Final weight (kg)	14.6	15.8	15.6	16.2	0.32	0.037	0.013	0.357
Average daily gain (g/d)	247	294	286	300	9.43	0.023	0.003	0.104
Average daily feed intake (g/d)	471	516	484	530	13.1	0.292	0.002	0.989
Feed/Gain (g/g)	1.91	1.76	1.71	1.77	0.05	0.087	0.459	0.057
Diarrhea rate	0.179	0.169	0.161	0.129	–	–	–	–
Mortality	0.2	0.1	0.1	0.0	–	–	–	–

*Piglets fed basal diet and orally administered 1 mL of 0.85% saline (CON); piglets fed basal diet and orally administered 1 mL 1 × 10^9^ cfu/mL C. utilis in 0.85% saline (CU); piglets fed the basal diet containing YSE (120 mg/kg) and orally administered 1 mL of 0.85% saline (YSE); Piglets fed the basal diet containing 120 mg/kg YSE and 1 mL 1 × 10^9^ cfu/mL C. utilis in 0.85% saline (YSE+CU). P < 0.05 means the difference is significant, P > 0.05 means the difference is not significant. The following tables and figures were the same as Table 2*.

### Effects of Dietary YSE and Oral CU on Organ Indexes of Weaned Piglets

As shown in Table 3, piglets dietary administered YSE increased the spleen index and lymph node index (*P* < 0.05), had a tend to increase the pancreatic index (*P* = 0.07), had no significant effect on cardiac, liver, thymus, kidney, pancreas indexes (*P* > 0.05). Piglets orally administered CU tended to increase spleen index (*P* = 0.05) and lymph node index (*P* = 0.08) and had no significant effect on cardiac, liver, thymus, kidney, pancreas indexes (*P* > 0.05). There were significant interaction effects on spleen index and lymph node index between YSE and CU (*P* < 0.05), a significant trend on liver index (*P* < 0.05), and no interaction effect on cardiac, liver, thymus, kidney and pancreas indexes (*P*> 0.05).

**Table 3:** Effects of dietary YSE and oral CU on organ indexes in weaned piglets (g/kg).

**Items**	**Treatments**				**SEM**	***P*****-value**		
	**CON**	**CU**	**YSE**	**CU+YSE**		**YSE**	**CU**	**CU × YSE**
Cardiac index	4.37	4.19	4.04	4.45	0.42	0.93	0.78	0.49
Liver index	25.6	24.9	26.3	27.2	1.95	0.55	0.29	0.06
Thymus index	0.42	0.63	0.51	0.54	0.13	0.86	0.38	0.52
Kidney index	5.26	5.79	5.55	6.14	0.31	0.32	0.86	0.12
Spleen index	1.68	1.95	2.06	2.96	0.21	< 0.05	0.05	0.005
Pancreas index	1.16	1.29	1.57	1.48	0.15	0.07	0.88	0.50
Mesenteric lymph nodes index	1.38	1.79	2.35	2.84	0.12	<0.05	0.08	0.03

### Effects of Dietary YSE and Oral CU on Gastrointestinal pH of Weaned Piglets

As shown in Table 4, piglets dietary administered YSE reduced rectal pH and urine pH (*P* < 0.05), and tended to decrease colon pH (*P* = 0.09), and had no significant effect on jejunum pH, ileum pH, and cecal pH (*P* > 0.05). Piglets orally administered CU reduced urine pH (*P* < 0.05) and had no significant effect on stomach pH, jejunum pH, ileum pH, colon pH, cecal pH, and rectal pH (*P* > 0.05). There were interaction effects on gastric pH, ileal pH, cecal pH, and urine pH between CU and YSE (*P* < 0.05), and there were no the interaction effects on jejunum pH, colon pH and cecal pH (*P* > 0.05).

**Table 4:** Effects of dietary YSE and oral CU on gastrointestinal tract pH in weaned piglets.

**Items (digesta contents)**	**Treatments**				**SEM**	***P*****-value**		
	**CON**	**CU**	**YSE**	**CU+YSE**		**YSE**	**CU**	**CU × YSE**
Stomach pH	3.37	3.40	2.93	2.85	0.42	0.33	0.28	0.01
Jejunum pH	5.71	5.86	5.73	5.39	0.32	0.49	0.77	0.46
Ileum pH	6.28	6.70	6.51	5.96	0.21	0.24	0.76	0.04
Colon pH	6.17	6.12	5.71	5.85	0.20	0.09	0.84	0.66
Cecum pH	5.84	6.11	6.00	5.80	0.17	0.65	0.85	0.19
Rectum pH	6.32	6.92	6.02	5.80	0.16	<0.01	0.25	0.02
Urine pH	7.36	6.66	6.44	6.32	0.13	0.05	0.03	0.06

### Effects of Dietary YSE and Oral CU on Plasma Biochemical Indexes of Weaned Piglets

As shown in Table 5, dietary supplementation of YSE increased serum GLU and BUN concentrations, serum T-SOD, T-AOC, CAT activity (*P* < 0.05), and decreased serum BUN and MDA concentrations (*P* < 0.05), and had no effect on serum T-CHO concentration, serum AST, ALT and A-CHE activity (*P* > 0.05). Piglets orally administered CU decreased serum MDA concentration (*P* < 0.05), increased serum T-SOD, T-AOC and CAT activity (*P* < 0.05), and had no significant effect on serum GLU, T-CHO concentration, serum AST, ALT, A-CHE activity (*P* < 0.05). Piglets dietary administered YSE and oral administered CU had significant interaction effects on serum T-SOD, T-AOC, and CAT activity and serum MDA concentration (*P* < 0.05), and had a decrease trend on serum BUN concentration (*P* = 0.06), no the interaction effect on serum GLU and T-CHO concentration, and serum T-SOD, AST, ALT and A-CHE activity (*P* > 0.05).

**Table 5:** Effects of dietary YSE and oral CU on serum parameters in weaned piglets.

**Items**	**Treatments**				**SEM**	***P*-value**			
	**CON**	**CU**	**YSE**	**CU+YSE**		**YSE**	**CU**	**CU × YSE**
GLU (mmol/L)	5.19	5.52	4.78	5.06	0.23	0.05	0.54	0.21
T-CHO (mmol/L)	2.27	2.12	2.07	2.06	0.16	0.15	0.23	0.75
AST (U/L)	28.5	28.9	27.3	25.4	1.69	0.25	0.32	0.18
ALT (U/L)	26.3	25.1	24.6	25.7	1.78	0.11	0.44	0.90
BUN (mmol/L)	4.73	4.54	3.15	3.77	0.20	<0.001	0.30	0.06
T-SOD (U/mgprot)	30.9	49.5	47.3	55.3	2.41	<0.001	0.04	<0.01
T-AOC (U/mL)	1.84	2.00	2.84	3.42	0.10	<0.01	<0.01	<0.01
MDA (nmol/L)	8.31	3.79	4.35	1.30	0.75	0.02	0.01	0.01
CAT (U/mL)	4.47	8.47	13.6	16.8	0.53	<0.01	<0.01	0.01
A-CHE (U/mL)	7.74	8.78	8.93	9.09	0.44	0.19	0.28	0.43

### Effects of Dietary YSE and Oral CU on Morphology and Structure of Ileal Mucosa in Weaned Piglets

**Figure 1:**
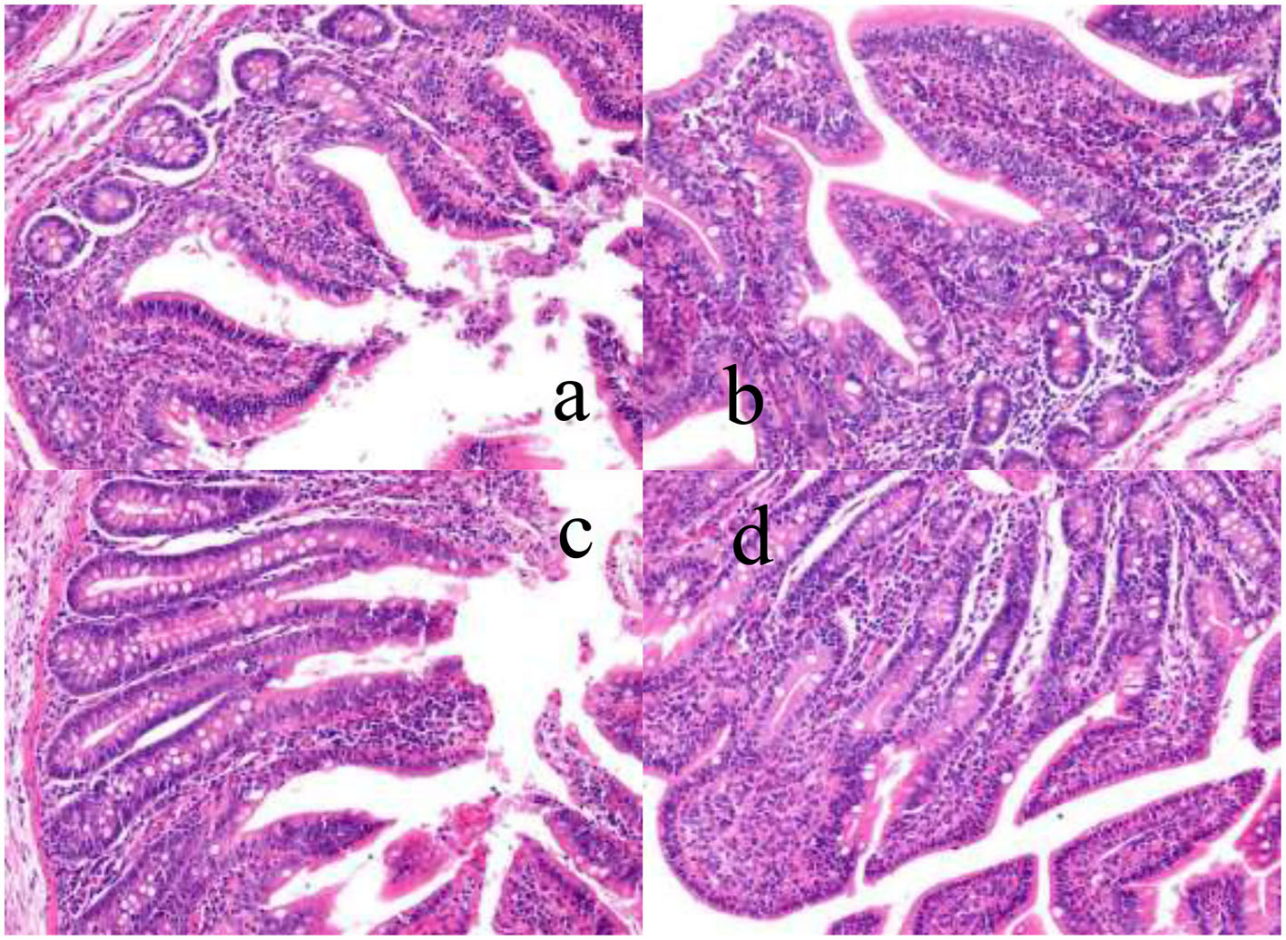
The effects of dietary YSE and oral CU on villus height and crypt depth of ileum in weaned pigs ( ×400). **(a)** represents piglets fed basal diet and orally administered 1 mL of 0.85% saline (CON), **(b)** represents piglets fed basal diet and orally administered 1 mL 1 × 10^9^ cfu/mL *C. utilis* in 0.85% saline (CU), **(c)** represents piglets fed the basal diet containing YSE (120 mg/kg) and orally administered 1 mL of 0.85% saline (YSE), **(d)** represents Piglets fed the basal diet containing 120 mg/kg YSE and 1 mL 1 × 10^9^ cfu/mL *C. utilis* in 0.85% saline (YSE+CU).

As shown in Figure 1, the structure of ileal villi of piglets in the control group was incomplete, there was shedding linearity, and the villi were sparsely arranged. Intestinal morphology and structure were not fully recovered, but the development of ileal villus structure of the three experimental groups was better than that of the control group, and the mucosal development was improved in varying degrees.

As shown in Table 6, dietary supplementation of YSE increased ileal villus height, villus height/crypt depth (*P* < 0.05), and decreased crypt depth (*P* < 0.05). Piglets orally administered CU increased ileal villus height and villus height/crypt depth (*P* < 0.05), tended to increase villus height (*P* <0.10), and decreased crypt depth (*P* < 0.05). There were significant interaction effects on between YSE and CU was significant in crypt depth, villus height/crypt depth (*P* <0.01), and trended to increase villus height (*P* = 0.08).

**Table 6:** Effects of dietary YSE and oral CU on morphology of ileum in weaned piglets.

**Items**	**Treatments**				**SEM**	***P*****-value**		
	**CON**	**CU**	**YSE**	**CU+YSE**		**YSE**	**CU**	**CU × YSE**
Villus height (μm)	471	484	497	516	9.42	0.01	0.10	0.08
Crypt depth (μm)	274	256	266	230	7.82	0.05	0.004	0.03
Vilius height/crypt depth	1.72	1.89	1.87	2.25	0.04	0.001	<0.001	0.03

### Effects of Dietary YSE and Oral CU on the Levels of β-definsin-2 and Occludin in Intestinal Mucosa of Weaned Piglets

**Figure 2:**
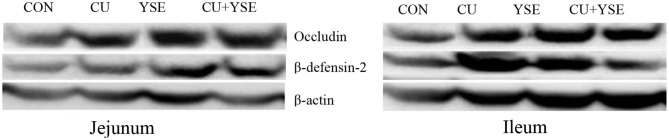
The effect of dietary YSE and oral CU on level of intestinal mucosa β-definsin-2 and occludin concentrations. Piglets fed basal diet and orally administered 1 mL of 0.85% saline (CON); piglets fed basal diet and orally administered 1 mL 1 × 10^9^ cfu/mL *C. utilis* in 0.85% saline (CU); piglets fed the basal diet containing YSE (120 mg/kg) and orally administered 1 mL of 0.85% saline (YSE); Piglets fed the basal diet containing 120 mg/kg YSE and 1 mL 1 × 10^9^ cfu/mL *C. utilis* in 0.85% saline (YSE+CU).

Figure 2 and Table 7 showed that dietary supplementation of YSE increased jejunal occludin, β-definsin-2 and ileal occludin concentration (*P* < 0.05), but had no significant effect on ileal β-definsin-2 levels (*P* > 0.05). Oral administration of CU increased the levels of occludin and β-definsin-2 in jejunum (*P* < 0.05), and had no significant effect on the concentration of occludin and β-definsin-2 in ileal mucosa (*P* > 0.05). There was the interaction effect on the concentration of occludin in jejunal mucosa between YSE and CU (*P* < 0.05), but not at the level of β-definsin-2 in jejunal and ileal mucosa (*P* > 0.05).

**Table 7:** Effects of dietary YSE and oral CU on level of intestinal mucosa β-definsin-2 and occludin concentration.

**Items**	**Treatments**				**SEM**	***P*****-value**		
	**CON**	**CU**	**YSE**	**CU+YSE**		**YSE**	**CU**	**CU × YSE**
**Jejunum**	
Occludin	0.34	0.43	0.57	0.76	0.03	<0.01	<0.01	0.10
β-definsin-2	0.89	1.04	1.07	1.31	0.09	0.02	0.04	0.63
**Ileum**	
Occludin	0.69	0.82	0.97	0.94	0.05	<0.01	0.15	<0.01
β-definsin-2	1.10	1.29	1.33	1.38	0.11	0.27	0.49	0.32

### Effects of Dietary YSE and Oral CU on Cecal Microflora of Weaned Piglets

**Figure 3:**
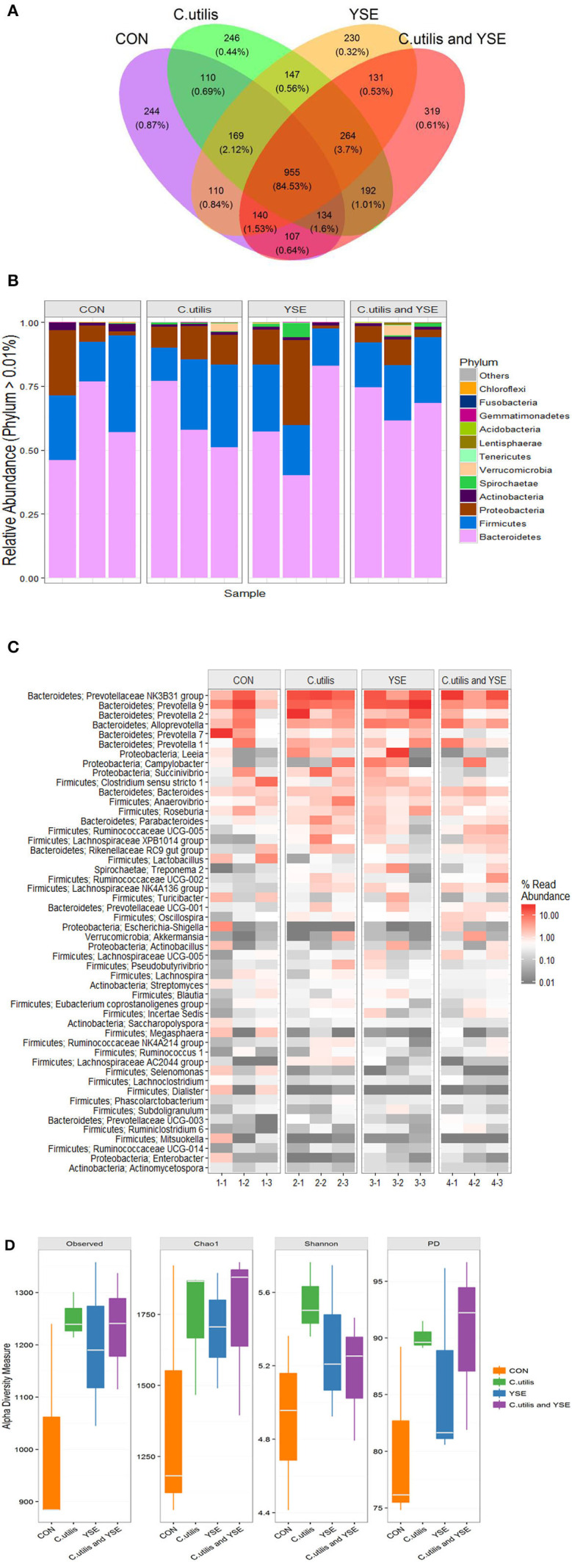
Effects of dietary YSE and oral CU on cecal microflora of weaned piglet. **(A)** The Ven pot; **(B)** phylum barplot; **(C)** genus heatmap; **(D)** Alpha diversity analysis; piglets fed basal diet and orally administered 1 mL of 0.85% saline (CON); piglets fed basal diet and orally administered 1 mL 1 × 10^9^ cfu/mL *C. utilis* in 0.85% saline (*C. utilis*); piglets fed the basal diet containing YSE (120 mg/kg) and orally administered 1 mL of 0.85% saline (YSE); Piglets fed the basal diet containing 120 mg/kg YSE and 1 mL 1 × 10^9^ cfu/mL *C. utilis* in 0.85% saline (*C. utilis* and YSE).

A total of 688,961 sequences were obtained from 12 samples in 4 groups by Miseq sequencing. After filtering some low abundance sequence, 566,122 sequences with 308 bp the average length were clustered into 3,498 OTU. These bacteria were divided into 21 phyla, 38 classes, 72 orders, 116 families, 294 genera, and 305 species. A Venn diagram was used to explore similarities and differences in microbial communities among groups, showing that the intestinal microbial communities in the caecum contents of piglets in the four groups had 959 common OTUs, accounting for 84.53% (Figure 3A). There were 244 specific OTUs in the control group, accounting for 0.87%, 246 specific OTU in the CU group, 230 specific OTUs in the YSE group, 319 specific OTUs in CU+YSE group.

Table 8 and Figure 3B showed dietary supplementation of YSE decreased the relative abundance of acidobacteria in cecum of weaned piglets (*P* < 0.05), the relative abundance of Spirochaetae had an increasing trend (*P* = 0.072). However, it had no significant effect on the relative abundance of Bacteroides, Firmicutes, Proteobacteria, Actinobacteria, Verrucomicrobia, Tenericutes, and Lentisphaerae (*P* > *0.05*). Oral administration of CU decreased the relative abundance of Proteobacteria and Actinobacteria (*P* < 0.05), and increased the relative abundance of Verrucomicrobia microbacteria, but had no effects on the relative abundance of Bacteroides, Firmicutes, Spirochaetae, Tenericutes, Lentisphaerae, and Acidobacteria (*P* > 0.05). There was no an interaction effect on the relative abundance of Proteus bacteria between YSE and CU, and decreased the relative abundance of Proteus bacteria (*P* < 0.05). YSE and CU had certain effects on the structure of cecal microflora of weaned piglets (*P* < 0.05).

**Table 8:** Effects of dietary YSE and oral CU on the relative abundance of caecum microflora at phylum level.

**Items**	**Treatments**				**SEM**	***P*****-value**		
	**CON**	**CU**	**YSE**	**CU+YSE**		**YSE**	**CU**	**CU × YSE**
Bacteroides	59.5	61.6	60.2	68.1	4.75	0.456	0.354	0.605
Firmicutes	26.2	24.3	20.0	21.8	3.78	0.283	0.988	0.638
Proteobacteria	11.2	10.9	15.5	6.40	1.31	0.947	0.007	0.01
Actinobacteria	2.30	1.05	1.22	1.05	0.32	0.126	0.055	0.126
Spirochaetae	0.07	0.43	2.20	0.60	0.55	0.072	0.295	0.115
Verrucomicrobia	0.04	0.94	0.06	1.43	0.14	0.117	<0.001	0.137
Tenericutes	0.03	0.11	0.12	0.11	0.03	0.19	0.317	0.189
Lentisphaerae	0.03	0.03	0.01	0.30	0.14	0.343	0.28	0.363
Acidobacteria	0.07	0.06	0.03	0.04	0.01	0.024	0.593	0.23

Table 9 and Figure 3C showed that the relative abundance of bacteria at the cecal microflora level of weaned piglets. Dietary supplementation of YSE increased the relative abundance of Bacteroides in cecum of weaned piglets (*P* < 0.05), and decreased the relative abundance of Clostridiaceae, Veillonellaceae, Erysipelotrichaceae, Acidaminococcaceae, Streptococcaceae, Campylobacteraceae, and Streptomycetaceae in weaned piglets (*P* < 0.05). It had no significant effect on the relative abundance of bacteria in Prevotellaceae, Porphyromonadaceae, Rikenellaceae, Cytophagaceae, Ruminococcaceae, Lactobacillaceae, and so on (*P* > 0.05). Oral administration of CU increased the relative abundance of Bacteroidales, Lachnospiraceae, and Ruminococcaceae (*P* < 0.05), and significantly decreased the relative abundance of Clostridiaceae, Streptococcaceae, Enterobacteriaceae, and Streptomycetaceae (*P* < 0.05). There no significant effect on the relative abundance of bacteria in Prevotellaceae, Porphyromonadaceae, Rikenellaceae, and Cytophagaceae (*P* > 0.05). YSE and CU had significant interaction effects on the relative abundance of Bacteroidales, Lachnospiraceae, Erysipelotrichaceae, Streptococcaceae, Campylobacteraceae, Enterobacteriaceae, Succinivibrionaceae, and Streptomycetaceae (*P* < 0.05).

**Table 9:** Effects of dietary YSE and oral CU on the relative abundance of caecum microflora at family level.

**Items**	**Treatments**				**SEM**	***P*****-value**		
	**CON**	**CU**	**YSE**	**CU+YSE**		**YSE**	**CU**	**CU × YSE**
Prevotellaceae	47.4	48.6	51.4	46	3.74	0.888	0.511	0.327
Bacteroidales	8.81	8.61	5.65	19.8	1.09	0.006	<0.001	<0.001
Porphyromonadaceae	0.72	0.90	0.89	0.89	0.18	0.479	0.228	0.689
Rikenellaceae	1.81	1.24	1.41	1.00	0.19	0.372	0.100	0.295
Cytophagaceae	0.15	0.17	0.13	0.14	0.02	0.396	0.470	0.739
Lachnospiraceae	6.09	10.3	7.84	8.11	0.67	0.746	0.010	0.018
Ruminococcaceae	4.90	6.46	5.42	7.37	0.61	0.738	0.006	0.287
Clostridiaceae	2.00	1.09	1.34	0.75	0.22	0.049	0.008	0.485
Veillonellaceae	4.76	2.48	1.43	1.63	0.50	0.001	0.311	0.177
Erysipelotrichaceae	1.78	0.51	0.55	0.32	0.13	0.001	<0.001	0.004
Lactobacillaceae	1.36	1.15	1.05	1.27	0.11	0.425	0.972	0.099
Peptostreptococcaceae	0.66	0.54	0.74	0.50	0.09	0.771	0.079	0.559
Acidaminococcaceae	0.42	0.40	0.28	0.30	0.04	0.028	0.943	0.794
Streptococcaceae	0.23	0.04	0.05	0.04	0.03	0.030	0.009	0.020
Neisseriaceae	0.76	0.63	0.68	0.62	0.11	0.683	0.439	0.756
Campylobacteraceae	0.44	0.25	0.13	0.17	0.05	0.003	0.156	0.039
Enterobacteriaceae	0.65	0.13	0.30	0.54	0.05	0.508	0.014	<0.001
Succinivibrionaceae	0.48	1.22	1.56	0.18	0.16	0.897	0.084	<0.001
Alcaligenaceae	0.21	0.17	0.19	0.14	0.04	0.560	0.330	0.917
Comamonadaceae	0.16	0.13	0.14	0.12	0.02	0.297	0.095	0.892
Streptomycetaceae	0.65	0.40	0.44	0.43	0.04	0.042	0.010	0.043

Table 10 and Figure 3D showed that compared with the control group, the Observed species, Chao1 and Fisher indexes of YSE group, CU group and mixed group were significantly higher than those of the control group (*P* < 0.05). The Shannon index in the YSE group and CU group was significantly higher than that in the control group (*P* < 0.05), but there was no significant difference between the mixed group and the control group (*P* > 0.05). There was no significant difference in PD index between CU group and mixed group (*P* > 0.05), but it was significantly higher than that in YSE group and control group (*P* < 0.05), and that in YSE group was significantly higher than that in control group (*P* < 0.05).

**Table 10:** Alpha diversity analysis.

**Items**	**Treatments**				**SEM**	***P*****-value**		
	**CON**	**CU**	**YSE**	**CU+YSE**		**YSE**	**CU**	**CU × YSE**
Observed	1003	1251	1197	1231	39.5	0.058	0.007	0.026
Chao1	1356	1735	1698	1737	61.2	0.023	0.009	0.024
Shannon	4.91	5.54	5.29	5.17	0.11	0.967	0.059	0.011
Simpson	0.95	0.98	0.97	0.96	0.01	0.86	0.729	0.146
Fisher	258	365	344	357	8.44	0.002	<0.001	0.001
Pd	80.0	90.0	86.1	90.3	0.88	0.008	<0.001	0.011

## Discussion

Our results showed that CU had a positive effect on the growth performance of weaned piglets. Oral active yeast preparation for early weaned piglets can help to relieve diarrhea, promote piglet growth, improve survival rate and growth performance (12). Active yeast preparation can promote the growth performance and enhance the immune function of piglets, and the mechanism of action may be related to the main components of cell wall, β-glucan, phosphorylated oligosaccharides and intracellular active peptides (13, 14). In this study, we fed CU together with its fermentation medium to piglets. Nucleotides, amino acids, peptides and other flavor substances in yeast culture were important factors to improve the feed intake of piglets. Yeast culture could significantly increase the feed intake of piglets (15). Yeast culture could improve ADG and feed utilization of piglets (16–18). Yeast culture stimulated intestinal fermentation, increased the yield of volatile fatty acids and the products of bacterial fermentation, and provided some energy for pigs to improve nutrient utilization (19–21).

Dietary 120 mg/kg YSE and oral CU in weaned piglets was 27.9% lower than that of the control group. YSE saponins reduce the concentration of ammonia and provide a good environment for digestion and absorption of nutrients. It is also the result of antioxidant and anti-inflammatory effects of YSE polyphenols. Some studied showed that YSE reduced the concentration of ammonia in livestock barn or the content of ammonia nitrogen in feces, but had no significant effect on growth performance (22, 23). This may be caused by different feed formula, environment, adding amount, physiological stage of pigs and so on. The feed composition, especially the content of crude protein, may affect the effect of YSE (24). Dietary 120 mg/kg YSE in weaned piglets also decreased the ammonia emission and improved growth performance (23). The addition amount of YSE in pig diets was different in different physiological stages. Generally speaking, the addition amount of YSE was 50–200 mg/kg.

The organ index can reflect the functional status of animals to a certain extent (25). Our results showed that oral CU tended to promote the development of spleen and mesenteric lymph nodes, and dietary YSE in diet promoted the spleen, pancreas and mesenteric lymph nodes. The specific mechanism of CU in promoting immune function may be related to the composition of cell wall. A certain proportion of yeast culture increased the concentration of intestinal IFN-γ in weaned piglets (15). YSE increased the immune organ index at low dose (100 and 200 mg/kg), but decreased the immune organ index at high dose (26).

Our results showed that piglets dietary administered YSE reduced rectal pH and urine pH significantly, piglets orally administered CU reduced urine pH and piglets dietary administered YSE and orally administered CU decreased gastric pH, ileal pH, cecal pH and urine pH. Oral yeast preparation decreased the pH value of intestinal segment, especially hindgut segment (27–29). Probiotic yeast inhibited the growth of *Escherichia coli*, promote the proliferation of Bifidobacterium and Lactobacillus, and cause the increase of intestinal VFA content. Dietary YSE could reduce the pH value of rumen fluid of dairy cows (30).

Our study showed that dietary YSE decreased the content of plasma urea nitrogen, which was consistent with the results of previous studies (31). Dietary 750 mg/kg significantly increased the content of ALT in animals, but high doses of YSE may have side effects on animals (32). In this study, dietary 120 mg / kg of YES did not affect the content of ALT, which indicated that this addition was safe in weaned piglets. Our study results showed that the extract of CU could significantly improve the antioxidant capacity and the contents of plasma glucose, urea nitrogen and total cholesterol in weaned piglets. Dietary YSE significantly decreased the level of serum glucose in diabetic rats (33). The hypoglycemic function of YSE is related to its saponins (34–36). In this study, dietary supplementation of YSE decreased plasma MDA concentration and increased plasma SOD concentration, which may be related to the scavenging effect of YSE on superoxide free radicals or preventing the formation of superoxides or peroxides (37). Our study showed that CU increased T-SOD activity, CAT activity, TmurAOC, and decreased MDA content, indicating that CU may have more potential in improving animal antioxidant capacity, which may be related to the ability of CU to synthesize glutathione (38, 39). Among all the experimental groups, CU and YSE synergistically improved the antioxidant capacity of weaned piglets, and the mixed group improved the antioxidant capacity of weaned piglets most obviously.

Villus height, crypt depth and villus height / crypt depth are important indexes to evaluate the absorption function of small intestine. Our results showed that oral CU and diet supplemented with YSE promoted the development of ileal mucosa, and they synergistically increased villus height / crypt depth. Under the physiological state, the villous epithelial cells fall off normally. The exfoliated cells migrate, differentiate and produce mature villus cells from the base of the crypt to the villus end (40). The depth of crypt becomes shallower, the maturation rate of epithelial cells increases, and the absorptive capacity of villi increases. Qi et al. (41) found that dietary YSE in piglets to reduce the concentration of ammonia nitrogen in the contents of duodenum, jejunum, ileum, cecum and colon, improve the intestinal villus structure of piglets, increase the villus width of jejunum, increase the villus length and width of jejunum and the ratio of villus length to crypt depth of jejunum. Yeast culture was used as a foreign antigen to stimulate the development of intestinal tract. Some studies have shown that the addition of active yeast or yeast and its culture to the diet is beneficial to the development of intestinal mucosa and promote intestinal health (42, 43). In this study, it was found that YSE and CU increased the villus height of ileal mucosa, villus height/crypt depth, and decreased the crypt depth, indicating that YSE and CU can alleviate intestinal health problems caused by weaning stress. This is also one of the reasonable explanations for the reduction of diarrhea rate in the above study.

Our study showed that oral CU and dietary YSE increased Occludin concentration in jejunum and ileum and the β-definsin-2 concentration in jejunum, and increased the level of occludin in jejunum and ileum. The main mode of connection between intestinal mucosal epithelial cells is tight junction to maintain the integrity of the mechanical structure and function of the intestinal mucosal barrier. Tight junctions are composed of peripheral cytoplasmic proteins such as occludin, claudins, junction adhesion molecules, and closed small ring proteins, in which occludin is a transmembrane protein related to the integrity of tight junctions. Studies have found that probiotics inhibit the translocation of pathogenic bacteria on the intestinal surface by maintaining the integrity of intestinal mucosa and reducing intestinal mucosal permeability, preventing toxins and harmful substances from entering the blood circulation, and pathogens didn't further invade the body (44). Oral administration of Saccharomyces cerevisiae induced specific immune response in mice infected with Clostridium, significantly increased the level of intestinal mucosal IgA alleviated diarrhea and intestinal mucosal damage induced by endotoxin produced by Clostridium, indicating that probiotics such as yeast can enhance intestinal immune response and maintain intestinal health (45). The possible mechanisms of probiotics protecting intestinal mucosal barrier are: maintaining the balance of intestinal flora, protecting microbial barrier, promoting mucus secretion, promoting the expression of tight junction protein, strengthening intestinal mucosal mechanical barrier, and stimulating intestinal mucosal immune response and inhibition of intestinal epithelial cell apoptosis (46).

The Venn diagram is used to reflect the common and unique species among the samples. Our research showed that the four groups accounted for 84.53% of the total OTU, while the unique OTU control group, CU group, YSE group and CU+YSE group were 0.87, 0.44, 0.32, and 0.61%, respectively, indicating that the experimental treatment had little effect on the core species in the intestinal tract of weaned piglets. The intestinal microflora of piglets has been changing since birth until a stable top community is formed (47). The proportion of OTU shared by the four groups reached 84.63%, which may be related to the stable top community gradually formed after weaning.

Our research showed that at the gate level, the cecal microorganisms of weaned piglets were mainly composed of Bacteroides and Firmicutes, which was consistent with the results of previous studies (48, 49). YSE and CU had no significant effect on the relative abundance of Bacteroides and Firmicutes bacteria, indicating that the addition of them had no significant effect on the dominant flora of weaned piglets. Proteobacteria are gram-negative bacteria, and many of them are pathogenic bacteria (50). Our results also showed that the YSE had no significant effect on the relative abundance of Proteus, but CU significantly reduced the relative abundance of Proteus, and the effect was better than that of CU alone. Acidobacteria are related to denitrification and nitrogen metabolism, and are greatly affected by nitrogen sources and pH (51). Our results showed that the YSE decreased the relative abundance of the bacteria, which may be related to the decrease of ammonia in the intestine. Bacteroides can ferment glucose, fructose, galactose, lactose, sucrose, and dextrin to produce acid and gas, resulting in a waste of energy. Our study showed that the increase of the relative abundance of Bacteroides was mainly caused by the interaction between YSE and CU, indicating that the interaction between YSE and CU was not ideal in improving the relative abundance of Bacteroides. Lachnospiraceae and Ruminococcaceae can ferment cellulose to produce butyric acid to inhibit colitis, so we usually think that they are beneficial bacteria (52). Our study showed that oral CU and its culture increased the relative abundance of Lachnospiraceae and Ruminococcaceae in weaned piglets, which was beneficial to the maintenance of intestinal health. We believe that the effect of yeast and its culture on increasing beneficial bacteria and reducing harmful bacteria may be related to oligosaccharides and other active components in yeast culture. These active substances maintain gastrointestinal microecological balance, selectively promote the proliferation of beneficial flora and inhibit the reproduction of harmful bacteria.

In addition, our study showed that the abundance and diversity of cecal microflora in weaned piglets increased with the addition of YSE or CU, alone or in combination. The increase of microbial diversity is usually positively correlated with the stability of microflora and the ability to resist the invasion of pathogens. Our results showed that dietary supplementation of YSE and CU could improve the diversity of cecal microflora and improve the health level of weaned piglets. Beta diversity analysis can support this result. YSE and CU had a certain effect on the structure of intestinal microflora of weaned piglets, which changed the sample distance between the control group and the experimental group in Beta diversity analysis. With the addition of YSE or CU, the change of microflora developed in the same direction, which showed that the samples of the three experimental groups were gathered together. It is suggested that the effects of YSE and CU on intestinal microorganisms may be consistent.

## Conclusion

This study provides evidence that YSE and CU are expected to replace antibiotics in the production of weaned piglets. Our results demonstrate that YSE and CU can improve the growth performance of weaned piglets, reduce the diarrhea rate of weaned piglets, improve intestinal health, and increase the diversity and richness of cecal microflora of weaned piglets. At the same time, we provided the specific information of YSE and CU on the growth performance and healthy development of weaned piglets, thus providing information for the development of feed plans that can improve or resist the stress of weaning piglets.

## Data Availability Statement

The original contributions presented in the study are included in the article/Supplementary material, further inquiries can be directed to the corresponding author/s.

## Ethics Statement

All experimental procedures in this study were approved by Sciences Animal Ethics Committee of Southwest University. Written informed consent was obtained from the owners for the participation of their animals in this study.

## Author Contributions

ZT and ZY designed the whole experiment. YW and TH performed the experiment, including chemical analysis, and statistical analysis. ZY, TH, and YW worked on the manuscript. ZS, GZ, LW, and WS verified the validity of experiment and checked the results. ZS, GZ, LW, and WS participated in the experiment design and gave valuable advice. All authors have read and approved the final version of this manuscript.

## Conflict of Interest

The authors declare that the research was conducted in the absence of any commercial or financial relationships that could be construed as a potential conflict of interest.
