# Supplementary material for: Effects of Dietary Yucca Schidigera Extract and Oral Candida utilis on Growth Performance and Intestinal Health of Weaned Piglets
Source: Front Nutr. 2021 May 26;8:685540. doi: 10.3389/fnut.2021.685540 (PMC8187599; doi:10.3389/fnut.2021.685540)
Supplement: Supplementary file 1 [file Data_Sheet_1.docx]

**Table 1** Ingredient composition of experimental diets in weaned piglets (g/kg, DM basis).

| Ingredients | 14%CP | 17%CP | 20%CP |
| --- | --- | --- | --- |
| Corn | 71.80 | 66.50 | 63.70 |
| Soybean meal | 13.40 | 18.80 | 19.80 |
| Whey powder | 4.40 | 4.30 | 4.30 |
| Fish meal | 1.50 | 4.00 | 9.00 |
| Soybean oil | 4.10 | 2.60 | 0.80 |
| L-Lys·HCl | 0.88 | 0.62 | 0.38 |
| DL-Met | 0.27 | 0.19 | 0.10 |
| L-Thr | 0.33 | 0.21 | 0.09 |
| L-Trp | 0.08 | 0.04 | 0.01 |
| Monocalcium phosphate | 1.15 | 0.74 | 0.00 |
| Limestone | 0.79 | 0.70 | 0.52 |
| Salt | 0.30 | 0.30 | 0.30 |
| Premix | 1.00 | 1.00 | 1.00 |
| Total | 100.00 | 100.00 | 100.00 |
| Nutritional level (Based on chemical analysis) | | | |
| DE（MJ/kg） | 14.60 | 14.60 | 14.60 |
| CP | 14.14 | 17.32 | 20.27 |
| Lys | 1.26 | 1.25 | 1.26 |
| Met+Cys | 0.63 | 0.65 | 0.62 |
| Thr | 0.76 | 0.75 | 0.76 |
| Trp | 0.20 | 0.20 | 0.20 |
| Arg | 0.71 | 0.93 | 1.09 |
| His | 0.30 | 0.37 | 0.44 |
| Ileu | 0.46 | 0.60 | 0.71 |
| Leu | 1.11 | 1.32 | 1.52 |
| Phe | 0.56 | 0.70 | 0.81 |
| Val | 0.54 | 0.64 | 0.72 |
| Ca, % | 0.70 | 0.71 | 0.69 |
| Available P, % | 0.53 | 0.55 | 0.57 |
| EAA | 6.29 | 7.18 | 7.91 |
| NEAA | 6.84 | 8.40 | 9.74 |
| EAA/NEAA | 0.90 | 0.85 | 0.80 |

**Note:** In addition to digestive energy data, the remaining nutrients are measured values.

**Table 2** Effects of low protein diet on nutrients excretion in feces (mg/g DM) and its apparent digestibility of piglets (%)

|  |  | DM-（%） | CP | CP-（%） | N | TN | TN-（%） | TPN | TPN-D (%) | TNN | TNN-D (%) | MTN | MPN | MNN |
| --- | --- | --- | --- | --- | --- | --- | --- | --- | --- | --- | --- | --- | --- | --- |
| d 10 | L-CP | 85.0 | 261 | 69.6 | 30.2 | 41.8 | 69.6 | 36.7 | 76.5 | 5.1 | 58.5 | 0.67 | 0.59 | 0.09 |
|  | M-CP | 83.7 | 282 | 70.9 | 29.1 | 45.1 | 70.9 | 40.1 | 74.3 | 5.0 | 54.7 | 1.34 | 1.23 | 0.21 |
|  | H-CP | 89.6 | 302 | 82.6 | 17.4 | 48.3 | 82.6 | 42.8 | 84.4 | 5.5 | 75.4 | 2.12 | 1.86 | 0.26 |
| d 25 | L-CP | 88.9 | 236 | 79.7 | 20.3 | 37.8 | 79.7 | 33.4 | 84.1 | 4.4 | 73.6 | 0.44 | 0.39 | 0.05 |
|  | M-CP | 88.8 | 271 | 80.8 | 19.2 | 43.4 | 80.8 | 38.0 | 83.5 | 5.4 | 66.0 | 0.94 | 0.84 | 0.11 |
|  | H-CP | 89.1 | 279 | 83.3 | 16.7 | 44.6 | 83.3 | 39.1 | 85.5 | 5.5 | 73.8 | 0.61 | 0.51 | 0.11 |
| d 45 | L-CP | 91.2 | 228 | 84.2 | 15.8 | 36.6 | 84.2 | 31.0 | 88.3 | 5.6 | 77.2 | 1.35 | 1.17 | 0.17 |
|  | M-CP | 88.6 | 208 | 84.1 | 15.9 | 33.3 | 84.1 | 28.1 | 88.4 | 5.2 | 75.6 | 1.86 | 1.67 | 0.19 |
|  | H-CP | 90.0 | 224 | 87.8 | 12.2 | 35.9 | 87.8 | 30.7 | 90.5 | 5.2 | 81.5 | 1.63 | 1.44 | 0.19 |
| SEM | Days | 0.38 | 3.0 | 0.59 | 0.59 | 0.48 | 0.59 | 0.47 | 0.49 | 0.07 | 0.63 | 0.018 | 0.015 | 0.005 |
|  | CP | 0.38 | 3.0 | 0.59 | 0.59 | 0.48 | 0.59 | 0.47 | 0.49 | 0.07 | 0.63 | 0.018 | 0.015 | 0.005 |
|  | Days×CP | 0.66 | 5.2 | 1.03 | 1.03 | 0.8 | 1.03 | 0.8 | 0.85 | 0.12 | 1.09 | 0.028 | 0.025 | 0.008 |
| P-Value | Days | <0.01 | <0.01 | <0.01 | <0.01 | <0.01 | <0.01 | <0.01 | <0.01 | 0.08 | <0.01 | <0.01 | <0.01 | <0.01 |
|  | CP | <0.01 | <0.01 | <0.01 | <0.01 | <0.01 | <0.01 | <0.01 | <0.01 | <0.01 | <0.01 | <0.01 | <0.01 | <0.01 |
|  | Days×CP | <0.01 | <0.01 | <0.01 | <0.01 | <0.01 | <0.01 | <0.01 | <0.01 | <0.01 | <0.01 | <0.01 | <0.01 | <0.01 |

**Note:** L-CP, Piglets fed a maize-soybean meal diet containing 14% CP; M-CP, Piglets fed a maize-soybean meal diet containing 17% CP; H-CP Piglets fed a maize-soybean meal diet containing 20% CP. SEM, standard error means. The same below.

**Table 3** Effects of low protein diet on total amino acids excretion in feces (mg/g DM) and its apparent digestibility (%) of piglets

|  |  | TAA  (+M) | TAA-D (+M) | NEAA  (+M) | NEAA-D (+M) | EAA  (+M) | EAA-D (+M) | E/NE  (+M) | TAA  (-M) | TAA-D (-M) | NEAA (-M) | NEAA-D (-M) | EAA (-M) | EAA-D  (-M) | EAA/NEAA  (-M) |
| --- | --- | --- | --- | --- | --- | --- | --- | --- | --- | --- | --- | --- | --- | --- | --- |
| d 10 | L-CP | 226 | 76.5 | 111 | 78.0 | 115 | 77.3 | 1.04 | 222.2 | 76.9 | 109.3 | 75.98 | 112.8 | 77.7 | 1.03 |
|  | M-CP | 251 | 74.3 | 120 | 76.5 | 131 | 74.4 | 1.09 | 243.8 | 75.1 | 117.5 | 74.91 | 126.6 | 75.2 | 1.08 |
|  | H-CP | 270 | 84.4 | 132 | 84.5 | 138 | 83.8 | 1.05 | 258.4 | 85.2 | 126.9 | 88.72 | 131.5 | 84.6 | 1.04 |
| d 25 | L-CP | 207 | 84.1 | 102 | 85.0 | 105 | 84.7 | 1.03 | 204.5 | 84.2 | 101.4 | 83.52 | 103.2 | 84.9 | 1.02 |
|  | M-CP | 236 | 83.5 | 117 | 84.3 | 119 | 84.1 | 1.02 | 231.2 | 83.9 | 114.3 | 83.31 | 116.3 | 84.4 | 1.02 |
|  | H-CP | 243 | 85.5 | 118 | 85.6 | 125 | 84.8 | 1.06 | 239.4 | 85.7 | 116 | 86.40 | 123.5 | 85.0 | 1.06 |
| d 45 | L-CP | 195 | 88.3 | 105 | 87.3 | 90 | 88.3 | 0.86 | 187.6 | 88.5 | 101.9 | 86.90 | 85.6 | 90.0 | 0.84 |
|  | M-CP | 179 | 88.4 | 87 | 88.8 | 92 | 89.2 | 1.06 | 169.0 | 88.1 | 82.3 | 87.83 | 86.7 | 88.3 | 1.05 |
|  | H-CP | 194 | 90.5 | 97 | 90.5 | 97 | 90.4 | 1.00 | 184.7 | 90.1 | 92.5 | 90.33 | 92.2 | 89.9 | 1.00 |
| SEM | Days | 3 | 0.49 | 1.4 | 0.49 | 1.70 | 0.50 | 0.06 | 3.0 | 0.48 | 1.7 | 0.49 | 1.8 | 0.48 | 0.07 |
|  | CP | 3 | 0.49 | 1.4 | 0.49 | 1.70 | 0.50 | 0.06 | 3.0 | 0.48 | 1.4 | 0.49 | 1.7 | 0.48 | 0.07 |
|  | Days×CP | 5.2 | 0.85 | 2.4 | 0.84 | 3 | 0.86 | 0.08 | 5.1 | 0.83 | 2.4 | 0.85 | 2.9 | 0.83 | 0.10 |
| *P*-Value | Days | <0.01 | <0.01 | <0.01 | <0.01 | <0.01 | <0.01 | <0.01 | <0.01 | <0.01 | <0.01 | <0.01 | <0.01 | <0.01 | <0.01 |
|  | CP | <0.01 | <0.01 | <0.01 | <0.01 | <0.01 | <0.01 | <0.01 | <0.01 | <0.01 | <0.01 | <0.01 | <0.01 | <0.01 | <0.01 |
|  | Days×CP | <0.01 | <0.01 | <0.01 | <0.01 | 0.06 | <0.01 | <0.01 | <0.01 | <0.01 | <0.01 | <0.01 | <0.01 | <0.01 | <0.01 |

**Table 4** Effects of low protein diet on amino acids excretion in feces of piglets (mg/g DM)

|  |  | Asp | Ser | Glu | Gly | Ala | Pro | Tyr | Thr | Cys | Val | Met | Ile | Leu | Phe | Lys | His | Arg |
| --- | --- | --- | --- | --- | --- | --- | --- | --- | --- | --- | --- | --- | --- | --- | --- | --- | --- | --- |
| d 10 | L-CP | 22.4 | 7.1 | 30.3 | 13.7 | 23.1 | 15.1 | 8.2 | 12.4 | 3.5 | 15.5 | 3.2 | 13.8 | 24.9 | 12.2 | 10.5 | 4.5 | 6.1 |
|  | M-CP | 23.0 | 8.3 | 32.7 | 17.3 | 24.7 | 14.3 | 9.0 | 11.6 | 3.8 | 18.8 | 4.4 | 16.4 | 29.1 | 14.2 | 12.0 | 4.6 | 7.2 |
|  | H-CP | 24.9 | 11.8 | 35.6 | 18.6 | 26.7 | 14.4 | 9.5 | 12.1 | 5.0 | 19.1 | 4.0 | 17.0 | 31.4 | 14.0 | 12.2 | 5.0 | 8.6 |
| d 25 | L-CP | 19.5 | 5.8 | 28.7 | 14.2 | 22.1 | 12.6 | 5.8 | 7.8 | 4.9 | 15.6 | 3.3 | 13.3 | 23.9 | 11.1 | 9.9 | 3.9 | 5.0 |
|  | M-CP | 22.6 | 7.6 | 31.6 | 16.9 | 24.8 | 13.5 | 7.7 | 10.2 | 3.7 | 17.7 | 3.5 | 15.5 | 26.4 | 11.9 | 11.4 | 4.2 | 6.5 |
|  | H-CP | 22.5 | 9.2 | 30.6 | 17.7 | 24.4 | 13.5 | 8.3 | 10.0 | 8.4 | 17.8 | 4.1 | 15.1 | 26.7 | 12.4 | 11.6 | 4.1 | 6.7 |
| d 45 | L-CP | 13.7 | 6.3 | 37.6 | 16.4 | 23.4 | 7.5 | 5.1 | 7.5 | 7.0 | 11.5 | 3.7 | 10.1 | 16.7 | 8.6 | 9.0 | 5.1 | 5.6 |
|  | M-CP | 15.9 | 5.9 | 22.8 | 13.4 | 19.7 | 9.4 | 6.1 | 7.5 | 3.6 | 14.3 | 2.2 | 12.1 | 20.5 | 9.9 | 8.1 | 3.4 | 4.5 |
|  | H-CP | 18.0 | 6.3 | 37.7 | 15.4 | 19.7 | 12.2 | 6.7 | 8.6 | 3.4 | 14.5 | 2.7 | 12.5 | 20.9 | 10.2 | 9.1 | 3.3 | 4.8 |
| SEM | Days | 0.4 | 0.2 | 0.5 | 0.3 | 0.3 | 0.3 | 0.2 | 0.2 | 0.4 | 0.2 | 0.1 | 0.3 | 0.5 | 0.2 | 0.2 | 0.1 | 0.2 |
|  | CP | 0.4 | 0.2 | 0.5 | 0.3 | 0.3 | 0.3 | 0.2 | 0.2 | 0.4 | 0.2 | 0.1 | 0.3 | 0.5 | 0.2 | 0.2 | 0.1 | 0.2 |
|  | Days×CP | 0.70 | 0.4 | 0.9 | 0.5 | 0.60 | 0.50 | 0.4 | 0.30 | 0.67 | 0.4 | 0.2 | 0.4 | 0.83 | 0.3 | 0.3 | 0.2 | 0.3 |
| *P*-Value | Days | <0.01 | <0.01 | <0.01 | <0.01 | <0.01 | <0.01 | <0.01 | <0.01 | 0.02 | <0.01 | <0.01 | <0.01 | <0.01 | <0.01 | <0.01 | <0.01 | <0.01 |
|  | CP | <0.01 | <0.01 | <0.01 | <0.01 | 0.33 | <0.01 | <0.01 | <0.01 | <0.01 | <0.01 | 0.33 | <0.01 | <0.01 | <0.01 | <0.01 | 0.05 | <0.01 |
|  | Days×CP | 0.23 | <0.01 | <0.01 | <0.01 | <0.01 | <0.01 | 0.6 | <0.01 | <0.01 | 0.46 | <0.01 | 0.57 | 0.25 | 0.58 | <0.01 | <0.01 | <0.01 |

**Table 5** Effects of low protein diet on amino acids apparent digestibility of piglets (%)

|  |  | Asp | Ser | Glu | Gly | Ala | Pro | Tyr | Thr | Cys | Val | Met | Ile | Leu | Phe | Lys | His | Arg |
| --- | --- | --- | --- | --- | --- | --- | --- | --- | --- | --- | --- | --- | --- | --- | --- | --- | --- | --- |
| d 10 | L-CP | 71.5 | 84.1 | 80.2 | 72.3 | 65.3 | 76.4 | 69.5 | 74.3 | 74.5 | 70.3 | 83.7 | 69.8 | 76.1 | 75.9 | 80.0 | 87.0 | 89.3 |
|  | M-CP | 76.7 | 79.8 | 79.0 | 64.2 | 60.8 | 77.1 | 64.4 | 74.0 | 69.9 | 61.3 | 92.1 | 62.7 | 68.8 | 70.4 | 76.4 | 86.0 | 87.2 |
|  | H-CP | 84.9 | 84.0 | 87.9 | 81.5 | 78.3 | 88.9 | 76.5 | 83.9 | 80.4 | 81.1 | 86.7 | 79.5 | 82.1 | 83.8 | 85.7 | 90.8 | 91.8 |
| d 25 | L-CP | 81.8 | 90.3 | 86.1 | 78.9 | 75.6 | 85.5 | 83.9 | 88.2 | 73.5 | 75.0 | 87.5 | 78.5 | 83.0 | 83.9 | 86.1 | 91.9 | 93.5 |
|  | M-CP | 84.3 | 87.3 | 86.0 | 76.0 | 73.3 | 85.3 | 79.1 | 84.3 | 79.6 | 87.3 | 95.7 | 75.9 | 80.7 | 83.0 | 84.6 | 91.2 | 92.1 |
|  | H-CP | 86.0 | 87.2 | 89.2 | 81.5 | 79.2 | 89.3 | 79.1 | 86.3 | 66.4 | 77.9 | 86.3 | 81.2 | 84.2 | 85.0 | 85.8 | 92.6 | 93.4 |
| d 45 | L-CP | 91.6 | 90.4 | 86.5 | 80.0 | 85.4 | 93.9 | 87.7 | 91.2 | 68.6 | 87.6 | 82.6 | 87.6 | 89.1 | 88.9 | 91.3 | 89.9 | 94.4 |
|  | M-CP | 89.0 | 90.0 | 91.2 | 83.5 | 84.2 | 91.4 | 84.7 | 89.3 | 83.2 | 83.1 | 92.7 | 83.6 | 86.7 | 87.7 | 91.2 | 92.8 | 95.1 |
|  | H-CP | 91.0 | 91.0 | 92.8 | 86.6 | 86.2 | 91.5 | 86.1 | 89.8 | 87.3 | 87.3 | 92.1 | 87.4 | 89.8 | 90.3 | 91.9 | 94.6 | 96.1 |
| SEM | Days | 0.59 | 0.5 | 0.43 | 0.65 | 0.77 | 0.5 | 0.85 | 0.64 | 1.69 | 0.68 | 0.65 | 0.74 | 0.56 | 0.58 | 0.47 | 0.44 | 0.25 |
|  | CP | 0.59 | 0.5 | 0.43 | 0.65 | 0.77 | 0.5 | 0.85 | 0.64 | 1.69 | 0.68 | 0.65 | 0.74 | 0.56 | 0.58 | 0.47 | 0.44 | 0.25 |
|  | Days×CP | 1.03 | 0.86 | 0.74 | 1.12 | 1.33 | 0.87 | 1.48 | 1.1 | 2.93 | 1.17 | 1.13 | 1.28 | 0.96 | 1.01 | 0.82 | 0.76 | 0.43 |
| *P*-Value | Days | <0.01 | <0.01 | <0.01 | <0.01 | <0.01 | <0.01 | <0.01 | <0.01 | 0.03 | <0.01 | 0.04 | <0.01 | <0.01 | <0.01 | <0.01 | <0.01 | <0.01 |
|  | CP | <0.01 | <0.01 | <0.01 | <0.01 | <0.01 | <0.01 | <0.01 | <0.01 | 0.03 | <0.01 | <0.01 | <0.01 | <0.01 | <0.01 | <0.01 | <0.01 | <0.01 |
|  | Days×CP | <0.01 | 0.04 | <0.01 | <0.01 | <0.01 | <0.01 | <0.01 | <0.01 | <0.01 | <0.01 | <0.01 | <0.01 | <0.01 | <0.01 | <0.01 | 0.01 | <0.01 |

**Table 6** Effects of low protein diet on the microbe amino acids excretion in feces of piglets (mg/g DM)

|  |  | Asp | Ser | Glu | Gly | Ala | Pro | Tyr | Thr | Cys | Val | Met | Ile | Leu | Phe | Lys | His | Arg | TAA | NEAA | EAA | EAA/NEAA |
| --- | --- | --- | --- | --- | --- | --- | --- | --- | --- | --- | --- | --- | --- | --- | --- | --- | --- | --- | --- | --- | --- | --- |
| 10d | L-CP | 0.315 | 0.145 | 0.435 | 0.216 | 0.376 | 0.173 | 0.165 | 0.156 | 0.025 | 0.320 | 0.058 | 0.260 | 0.312 | 0.255 | 0.205 | 0.108 | 0.168 | 3.70 | 1.66 | 2.03 | 1.22 |
|  | M-CP | 0.617 | 0.253 | 0.710 | 0.535 | 0.265 | 0.362 | 0.302 | 0.365 | 0.063 | 0.706 | 0.065 | 0.563 | 0.795 | 0.456 | 0.428 | 0.213 | 0.336 | 7.03 | 2.74 | 4.29 | 1.38 |
|  | H-CP | 0.765 | 0.438 | 1.340 | 0.712 | 1.406 | 0.516 | 0.565 | 0.690 | 0.052 | 0.985 | 0.310 | 0.728 | 1.011 | 0.683 | 0.626 | 0.265 | 0.522 | 11.61 | 5.19 | 6.43 | 1.32 |
| 25d | L-CP | 0.213 | 0.118 | 0.265 | 0.150 | 0.263 | 0.087 | 0.083 | 0.223 | 0.010 | 0.172 | 0.018 | 0.153 | 0.240 | 0.136 | 0.136 | 0.070 | 0.085 | 2.44 | 1.10 | 1.34 | 1.29 |
|  | M-CP | 0.680 | 0.155 | 0.658 | 0.346 | 0.670 | 0.168 | 0.150 | 0.268 | 0.020 | 0.297 | 0.097 | 0.335 | 0.498 | 0.266 | 0.280 | 0.135 | 0.232 | 5.25 | 2.67 | 2.58 | 1.25 |
|  | H-CP | 0.352 | 0.132 | 0.352 | 0.216 | 0.330 | 0.108 | 0.125 | 0.230 | 0.035 | 0.198 | 0.048 | 0.241 | 0.295 | 0.17 | 0.155 | 0.090 | 0.12 | 3.20 | 1.48 | 1.71 | 1.23 |
| 45d | L-CP | 0.698 | 0.343 | 0.792 | 0.633 | 0.628 | 0.176 | 0.395 | 0.462 | 0.052 | 0.348 | 0.160 | 0.677 | 0.395 | 0.593 | 0.467 | 0.270 | 0.346 | 7.35 | 3.27 | 5.08 | 1.25 |
|  | M-CP | 1.045 | 0.355 | 1.233 | 0.702 | 1.168 | 0.362 | 0.408 | 0.533 | 0.105 | 0.758 | 0.170 | 0.702 | 1.108 | 0.628 | 0.505 | 0.253 | 0.395 | 10.04 | 4.86 | 5.57 | 1.15 |
|  | H-CP | 0.853 | 0.351 | 1.212 | 0.532 | 1.030 | 0.308 | 0.350 | 0.470 | 0.077 | 0.593 | 0.153 | 0.545 | 1.070 | 0.553 | 0.405 | 0.187 | 0.312 | 9.00 | 4.29 | 4.72 | 1.10 |
| SEM | Days | 0.011 | 0.012 | 0.016 | 0.009 | 0.018 | 0.008 | 0.014 | 0.012 | 0.004 | 0.013 | 0.005 | 0.090 | 0.014 | 0.009 | 0.008 | 0.006 | 0.01 | 0.044 | 0.057 | 0.011 | 0.08 |
|  | CP | 0.011 | 0.012 | 0.016 | 0.009 | 0.018 | 0.008 | 0.014 | 0.012 | 0.004 | 0.013 | 0.005 | 0.090 | 0.014 | 0.009 | 0.008 | 0.006 | 0.01 | 0.044 | 0.057 | 0.011 | 0.08 |
|  | Days×CP | 0.020 | 0.020 | 0.027 | 0.016 | 0.032 | 0.014 | 0.024 | 0.021 | 0.007 | 0.022 | 0.009 | 0.016 | 0.024 | 0.016 | 0.015 | 0.011 | 0.017 | 0.161 | 0.075 | 0.098 | 0.10 |
| *P*-Value | Days | <0.01 | <0.01 | <0.01 | <0.01 | <0.01 | <0.01 | <0.01 | <0.01 | <0.01 | <0.01 | <0.01 | <0.01 | <0.01 | <0.01 | <0.01 | <0.01 | <0.01 | <0.01 | <0.01 | 0.04 | <0.01 |
|  | CP | <0.01 | <0.01 | <0.01 | <0.01 | <0.01 | <0.01 | <0.01 | <0.01 | <0.01 | <0.01 | <0.01 | <0.01 | <0.01 | <0.01 | <0.01 | <0.01 | <0.01 | <0.01 | <0.01 | <0.01 | <0.01 |
|  | Days×CP | <0.01 | <0.01 | <0.01 | <0.01 | <0.01 | <0.01 | <0.01 | <0.01 | 0.010 | <0.01 | <0.01 | <0.01 | <0.01 | <0.01 | <0.01 | <0.01 | <0.01 | <0.01 | <0.01 | <0.01 | <0.01 |

**Table 7** Effects of low protein diet on the microbial bacteria amino acids composition in feces of piglets (%)

|  |  | Asp | Ser | Glu | Gly | Ala | Pro | Tyr | Thr | Cys | Val | Met | Ile | Leu | Phe | Lys | His | Arg | NEAA | EAA | NEAA/EAA |
| --- | --- | --- | --- | --- | --- | --- | --- | --- | --- | --- | --- | --- | --- | --- | --- | --- | --- | --- | --- | --- | --- |
| 10d | L-CP | 8.56 | 3.93 | 11.76 | 5.87 | 10.25 | 4.62 | 4.36 | 4.27 | 0.67 | 8.67 | 1.55 | 7.050 | 8.52 | 6.92 | 5.57 | 2.90 | 4.54 | 45.0 | 55.0 | 1.22 |
|  | M-CP | 8.33 | 3.43 | 9.62 | 7.23 | 8.49 | 4.87 | 4.06 | 4.92 | 0.84 | 10.05 | 0.88 | 7.630 | 10.73 | 6.18 | 5.80 | 2.91 | 4.55 | 42.0 | 58.0 | 1.38 |
|  | H-CP | 6.60 | 3.80 | 11.54 | 6.15 | 12.13 | 4.45 | 4.88 | 5.94 | 0.44 | 8.31 | 2.69 | 6.300 | 8.72 | 5.89 | 5.41 | 2.30 | 4.47 | 44.7 | 55.3 | 1.32 |
| 25d | L-CP | 8.82 | 4.87 | 10.92 | 6.17 | 10.79 | 3.54 | 3.38 | 9.25 | 0.52 | 7.13 | 0.79 | 6.240 | 9.90 | 5.61 | 5.65 | 2.86 | 3.57 | 45.1 | 54.9 | 1.29 |
|  | M-CP | 8.93 | 3.41 | 10.90 | 7.56 | 9.19 | 3.67 | 3.16 | 5.89 | 0.44 | 6.44 | 2.05 | 7.330 | 10.98 | 5.90 | 6.12 | 2.93 | 5.09 | 43.7 | 56.3 | 1.25 |
|  | H-CP | 11.04 | 4.13 | 11.04 | 6.80 | 10.20 | 3.3 | 3.84 | 7.31 | 1.13 | 6.26 | 1.44 | 7.610 | 9.15 | 5.36 | 4.89 | 2.89 | 3.62 | 46.5 | 53.5 | 1.23 |
| 45d | L-CP | 9.54 | 4.64 | 10.79 | 8.62 | 8.53 | 2.4 | 5.38 | 6.27 | 0.72 | 4.73 | 0.99 | 9.180 | 5.33 | 8.09 | 6.38 | 3.69 | 4.72 | 44.5 | 55.5 | 1.25 |
|  | M-CP | 10.01 | 3.41 | 11.80 | 6.72 | 11.20 | 3.46 | 3.91 | 5.13 | 1.01 | 7.26 | 1.67 | 6.710 | 10.62 | 6.05 | 4.83 | 2.42 | 3.80 | 46.6 | 53.4 | 1.15 |
|  | H-CP | 9.49 | 3.91 | 13.47 | 5.89 | 11.46 | 3.42 | 3.88 | 5.20 | 0.85 | 6.58 | 1.70 | 6.070 | 11.92 | 6.15 | 4.51 | 2.06 | 3.45 | 47.6 | 52.4 | 1.10 |
| SEM | Days | 0.19 | 0.14 | 0.16 | 0.13 | 0.23 | 0.11 | 0.17 | 0.16 | 0.05 | 0.17 | 0.07 | 0.11 | 0.24 | 0.12 | 0.15 | 0.10 | 0.12 | 0.28 | 0.28 | 0.07 |
|  | CP | 0.19 | 0.14 | 0.16 | 0.13 | 0.23 | 0.11 | 0.17 | 0.16 | 0.05 | 0.17 | 0.07 | 0.11 | 0.24 | 0.12 | 0.15 | 0.10 | 0.12 | 0.28 | 0.28 | 0.07 |
|  | Days×CP | 0.32 | 0.24 | 0.27 | 0.23 | 0.40 | 0.19 | 0.30 | 0.28 | 0.09 | 0.29 | 0.11 | 0.19 | 0.41 | 0.20 | 0.20 | 0.17 | 0.21 | 0.49 | 0.49 | 0.09 |
| *P*-Value | Days | <0.01 | 0.12 | <0.01 | <0.01 | 0.59 | <0.01 | <0.01 | <0.01 | 0.02 | <0.01 | <0.01 | 0.09 | 0.06 | <0.01 | 0.20 | 0.32 | <0.01 | <0.01 | <0.01 | <0.01 |
|  | CP | 0.9 | <0.01 | <0.01 | <0.01 | <0.01 | 0.01 | 0.02 | <0.01 | 0.08 | <0.01 | <0.01 | <0.01 | <0.01 | <0.01 | <0.01 | <0.01 | <0.01 | <0.01 | <0.01 | <0.01 |
|  | Days×CP | <0.01 | 0.36 | <0.01 | <0.01 | <0.01 | <0.01 | <0.01 | <0.01 | <0.01 | <0.01 | <0.01 | <0.01 | <0.01 | <0.01 | <0.01 | <0.01 | <0.01 | <0.01 | <0.01 | <0.01 |

**Table 8** Effects of low protein diet on the microbial bacteria amino acids composition in ileum of piglets (%)

|  |  | Asp | Ser | Glu | Gly | Ala | Pro | Tyr | Thr | Cys | Val | Met | Ile | Leu | Phe | Lys | His | Arg | NEAA | EAA | EAA/NEAA |
| --- | --- | --- | --- | --- | --- | --- | --- | --- | --- | --- | --- | --- | --- | --- | --- | --- | --- | --- | --- | --- | --- |
| 10d | L-CP | 9.41 | 3.64 | 11.61 | 5.46 | 7.23 | 3.93 | 4.52 | 5.26 | 0.46 | 7.44 | 0.41 | 7.19 | 13.41 | 7.17 | 4.78 | 3.32 | 4.78 | 42.3 | 58.7 | 1.39 |
|  | M-CP | 9.89 | 3.39 | 11.47 | 7.05 | 6.42 | 3.88 | 4.11 | 5.64 | 0.46 | 6.70 | 0.28 | 6.69 | 12.59 | 7.33 | 5.47 | 3.46 | 5.19 | 42.1 | 57.9 | 1.38 |
|  | H-CP | 8.76 | 3.60 | 12.05 | 8.05 | 7.25 | 4.98 | 3.47 | 4.92 | 0.44 | 8.68 | 0.40 | 6.81 | 12.19 | 6.46 | 4.75 | 2.99 | 4.22 | 44.7 | 55.3 | 1.24 |
| 25d | L-CP | 9.44 | 4.65 | 10.23 | 5.52 | 5.96 | 5.48 | 4.09 | 4.87 | 0.48 | 6.47 | 0.68 | 6.41 | 13.71 | 8.14 | 3.86 | 4.67 | 5.35 | 41.3 | 58.7 | 1.42 |
|  | M-CP | 11.27 | 3.95 | 12.80 | 6.39 | 6.35 | 4.75 | 3.60 | 4.72 | 0.26 | 7.87 | 0.38 | 8.00 | 12.65 | 7.16 | 3.67 | 2.59 | 3.63 | 45.5 | 54.5 | 1.20 |
|  | H-CP | 10.72 | 4.76 | 12.58 | 7.23 | 6.06 | 7.26 | 2.91 | 4.93 | 0.27 | 7.40 | 0.22 | 6.73 | 12.31 | 7.46 | 4.02 | 2.77 | 2.38 | 48.6 | 51.4 | 1.06 |
| 45d | L-CP | 10.92 | 4.25 | 9.77 | 5.82 | 6.53 | 4.93 | 2.96 | 5.02 | 0.48 | 7.34 | 0.63 | 8.32 | 12.03 | 8.51 | 4.10 | 4.62 | 3.74 | 42.2 | 57.8 | 1.37 |
|  | M-CP | 10.37 | 5.43 | 11.96 | 6.43 | 7.07 | 4.57 | 3.04 | 4.67 | 0.55 | 6.28 | 0.35 | 6.27 | 12.60 | 6.46 | 4.82 | 4.37 | 3.14 | 47.5 | 52.5 | 1.11 |
|  | H-CP | 10.80 | 6.15 | 12.58 | 7.53 | 7.80 | 4.44 | 3.87 | 5.37 | 0.42 | 6.47 | 0.47 | 7.08 | 10.09 | 6.34 | 3.86 | 2.77 | 3.13 | 49.3 | 50.7 | 1.03 |
| SEM | Days | 0.15 | 0.11 | 0.22 | 0.16 | 0.14 | 0.12 | 0.12 | 0.12 | 0.02 | 0.21 | 0.01 | 0.12 | 0.18 | 0.13 | 0.09 | 0.09 | 0.08 | 0.30 | 0.30 | 0.02 |
|  | CP | 0.15 | 0.11 | 0.22 | 0.16 | 0.14 | 0.12 | 0.12 | 0.12 | 0.02 | 0.21 | 0.01 | 0.12 | 0.18 | 0.13 | 0.09 | 0.09 | 0.08 | 0.30 | 0.30 | 0.02 |
|  | Days×CP | 0.26 | 0.19 | 0.38 | 0.28 | 0.28 | 0.24 | 0.20 | 0.21 | 0.04 | 0.36 | 0.02 | 0.21 | 0.31 | 0.23 | 0.16 | 0.16 | 0.14 | 0.52 | 0.52 | 0.03 |
| *P*-Value | Days | <0.01 | <0.01 | 0.387 | 0.12 | <0.01 | <0.01 | <0.01 | 0.04 | <0.01 | 0.01 | <0.01 | 0.15 | 0.03 | 0.05 | <0.01 | <0.01 | <0.01 | <0.01 | <0.01 | <0.01 |
|  | CP | <0.01 | <0.01 | <0.01 | <0.01 | 0.04 | <0.01 | 0.03 | 0.93 | 0.01 | 0.14 | <0.01 | 0.04 | <0.01 | <0.01 | <0.01 | <0.01 | <0.01 | <0.01 | <0.01 | <0.01 |
|  | Days×CP | <0.01 | <0.01 | <0.01 | 0.35 | <0.01 | <0.01 | <0.01 | 0.03 | <0.01 | <0.01 | <0.01 | <0.01 | 0.06 | <0.01 | <0.01 | <0.01 | <0.01 | <0.01 | <0.01 | <0.01 |
